# Gut commensal *Bifidobacterium longum* confers resistance to *Salmonella* Typhimurium and *Shigella flexneri* in a *Caenorhabditis elegans* model

**DOI:** 10.1128/spectrum.01842-25

**Published:** 2025-12-05

**Authors:** Phurt Harnvoravongchai, Samara Paula Mattiello, Achuthan Amabat, Jusail C. P., Syed M. Faisal, Radhey S. Kaushik, Joy Scaria

**Affiliations:** 1 Department of Biology, Faculty of Science, Mahidol University 98842, Bangkok, Thailand; 2 Department of Veterinary Pathobiology, Oklahoma State University 316729, Stillwater, Oklahoma, USA; 3 Department of Veterinary and Biomedical Sciences, South Dakota State University 114635, Brookings, South Dakota, USA; 4 School of Mathematics and Sciences, University of Tennessee Southern 41289, Pulaski, Tennessee, USA; 5 Laboratory of Vaccine Immunology, National Institute of Animal Biotechnology 378803, Hyderabad, India; 6 Department of Biology and Microbiology, South Dakota State University 2019, Brookings, South Dakota, USA; Tainan Hospital Ministry of Health and Welfare, Tainan, Taiwan

**Keywords:** colonization resistance, gut microbiota, non-antibiotic therapy

## Abstract

Salmonellosis and shigellosis remain major global health concerns, with *Salmonella* Typhimurium and *Shigella flexneri* classified as high-priority antibiotic-resistant pathogens by the World Health Organization. The development of new antibiotics is slow and challenging, underscoring the need for alternative therapeutic strategies. One promising approach involves leveraging gut microbiota-derived bacteria that confer colonization resistance against enteric pathogens. In this study, we screened a human gut microbiota culture collection and identified *Bifidobacterium longum* as the most effective species in inhibiting *S*. Typhimurium and *S. flexneri in vitro*. To evaluate its protective potential *in vivo*, we utilized *Caenorhabditis elegans* as a model system. Our findings demonstrate that *B. longum* significantly reduced pathogen burden and enhanced host survival following infection. Mechanistic analysis revealed that *B. longum* inhibits *S*. Typhimurium primarily through acidification, while *S. flexneri* suppression appears to involve a protein-mediated or heat-stable metabolite-dependent mechanism. Additionally, *B. longum* modulated host immune pathways, downregulating genes associated with the p38 MAPK and insulin/IGF-1 signaling pathways. These results highlight the potential of *B. longum* as a non-antibiotic therapeutic for controlling *Salmonella* and *Shigella* infections. However, further validation in mammalian models is required to assess its clinical relevance.

**IMPORTANCE:**

Gut infections caused by *Salmonella* and *Shigella* are major global health threats. As an alternative to novel drug discovery, which is time-consuming and faces several challenges, this study explores the potential of gut bacteria to protect against these pathogens. We identified *Bifidobacterium longum*, a common gut microbe, which can significantly reduce infection by both *Salmonella* and *Shigella* in a lab setting and in a simple animal model. The bacterium functions by creating an environment that is hostile to pathogens and by modulating the host’s immune responses. These findings suggest that *B. longum* could be developed as a natural, non-antibiotic treatment to control or reduce these enteric pathogen infections. This approach opens the door to using probiotics as effective tools in the global fight against antibiotic resistance.

## INTRODUCTION

The rise in antibiotic resistance among human bacterial pathogens presents a significant global challenge. The World Health Organization’s (WHO) 2024 bacterial priority pathogen list includes 24 pathogens across 15 families of antibiotic-resistant bacteria, underscoring the escalating concern (1). Several pathogens, particularly those classified as critical and high priority, exhibit multidrug resistance. This issue is exacerbated by the lengthy development and approval process for new-generation antibiotics, which typically requires 10–15 years from discovery to market authorization. Moreover, the success rate for novel classes of antibiotics remains low, with only one in 30 preclinical trials progressing to approval (2).

As a result, there is an urgent need to develop non-antibiotic alternatives to control multidrug-resistant bacteria. In this regard, bacteria from healthy gut microbiota constitute a rich source of pathogen-suppressing species, primarily through a mechanism known as colonization resistance (3–5). They outcompete pathogens for nutrients and attachment (6, 7), produce antimicrobial compounds such as bacteriocins and short-chain fatty acids, enhance gut barrier function by regulating tight junction proteins (8), and modulate the immune responses to promote host defense while preventing excessive inflammation (9–11). The colonization resistance effect on the gut microbiota has been leveraged in fecal microbiota transplantation (FMT), where fecal material from healthy patients is transferred to patients with antibiotic-refractory diarrhea. While FMT is highly effective as a mainstream treatment for diarrheal infections, challenges in large-scale preparation could limit its application, particularly to severe cases (12, 13).

Identifying specific bacterial species responsible for colonization resistance is challenging due to the gut microbiota’s complexity and the interconnected role of its members (14, 15). Multiple bacterial species interact in intricate ways, making it difficult to isolate individual effects (16, 17). Some bacteria indirectly enhance immune defenses, further complicating the distinction between direct inhibition and immune-mediated resistance. Additionally, functional overlap among closely related commensals adds another layer of complexity. However, recent efforts have led to the development of gut microbiota culture collections to address these challenges (17–20). In this study, we utilized a culture collection (20) to screen and identify candidate species capable of inhibiting *Salmonella* Typhimurium and *Shigella flexneri*. These two pathogens were selected for screening because both are listed as high-priority antibiotic-resistant pathogens by the WHO (1). Non-typhoidal *Salmonella* causes approximately 93.8 million infections annually, representing a major global health and economic burden (21, 22). *S*. Typhimurium has a broad host range that extends beyond humans to include livestock such as chickens and pigs, which play a significant role in its transmission and the occurrence of widespread outbreaks globally (23, 24). While effective vaccines exist for typhoid fever, few options are available for non-typhoidal salmonellosis, despite its broad host range and impact on livestock. *Shigella* similarly causes over 80 million infections each year, with more than 69% of cases occurring in children under 5 years of age (25, 26). For our screen, we employed both *in vitro* assays and an *in vivo* model. We chose *Caenorhabditis elegans* as the *in vivo* model due to its simplicity and natural ability to consume bacteria. Numerous human gut pathogens, including gram-negative Diarrheagenic *Escherichia coli*, *Salmonella enterica*, *Vibrio cholerae*, and gram-positive *L. monocytogenes*, have been examined using this model (27–31). Alterations in worm behavior, motility, and innate immune signaling pathways are commonly employed to study bacterial pathogenesis and host responses (31, 32). This combined *in vitro*/*in vivo* approach enabled a rapid platform to screen microbiota strains for pathogen inhibition. Our screen identified *Bifidobacterium longum* as the most effective species against both *Salmonella* and *Shigella*. This strain also showed significant protection in the *C. elegans* model through innate immune modulation and enhanced host defense responses.

## RESULTS

### Screening and identification of bacterial species capable of inhibiting *Salmonella* and *Shigella*

**Fig 1:**
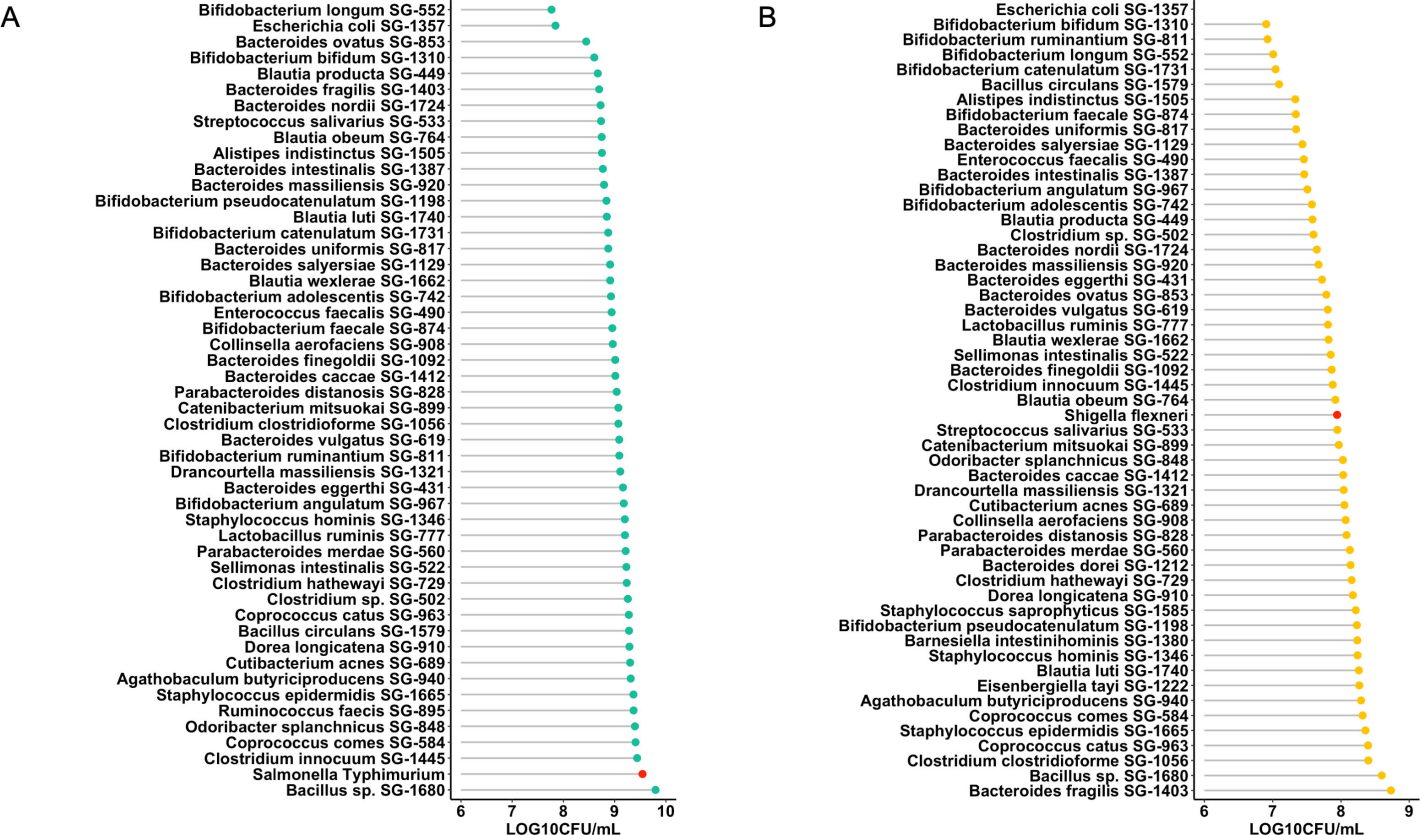
Screening of bacteria from human culture collection to identify species capable of inhibiting enteric pathogens. (**A**) Inhibitory effects of 49 species on *Salmonella* growth. (**B**) Inhibitory range of 52 species against *Shigella*. Co-cultures were incubated anaerobically for 24 h at a 9:1 ratio (bacteria:pathogen). The growth of the pathogen alone is indicated by a red dot in the lollipop plot. Values represent the mean from three independent experiments.

Our group previously developed a microbiota culture collection from healthy human donors that represented 70% of the functional capacity of the gut microbiota (20). We used this culture collection to identify species capable of inhibiting *S.* Typhimurium and *S. flexneri* growth. Since growth rate is a critical factor in competitive exclusion and fast-growing bacteria have strong potential for colonization resistance due to their efficiency in competing for nutrients and occupying available niche spaces, approximately 52 fast-growing species from this collection were chosen for this screen using co-culture assays. Most of the strains tested inhibited *Salmonella* to varying degrees, except for *Bacillus* sp. SG-1680, which promoted *Salmonella* growth (Fig. 1A). *B. longum* SG-552 and *E. coli* SG-1357 exhibited the most pronounced inhibitory effects with 1.77 and 1.70 log reductions, respectively. In the case of *Shigella*, inhibition was limited to 29 strains, whereas 23 species enhanced *Shigella* growth (Fig. 1B). *Bacteroides fragilis* SG-1403 exhibited the highest enhancement *of Shigella* growth, by nearly 1 log. In contrast, this species was the most effective at inhibiting *Salmonella*. The bacterial species exhibiting the most significant inhibitory effects on the pathogens: *B. longum* SG-552, *E. coli* SG-1357, *Bacteroides ovatus* SG-853, *Bifidobacterium bifidum* SG-1310, *Blautia producta* SG-449, *Bacteroides fragilis* SG-1403, *Bifidobacterium ruminantium* SG-811, *Bifidobacterium catenulatum* SG-1731, *Bacillus circulans* SG-1579, and *Alistipes indistinctus* SG-1505 were selected for further investigation. To assess the safety profile of these species, a cell invasion assay using human epithelial Caco-2 cells was performed. The results showed that *B. bifidum*, *B. circulans*, and *E. coli* actively invaded the Caco-2 cells. No invasion of Caco-2 cells by *A. indistinctus*, *B. catenulatum*, *B. fragilis*, *B. longum*, *B. ovatus*, and *B. producta* was observed, while relatively low invasion was observed for *B. ruminantium* (Fig. S1). Therefore, species that demonstrated substantial inhibitory effects with minimal cell invasion were chosen as candidates for subsequent experiments.

**Fig 2:**
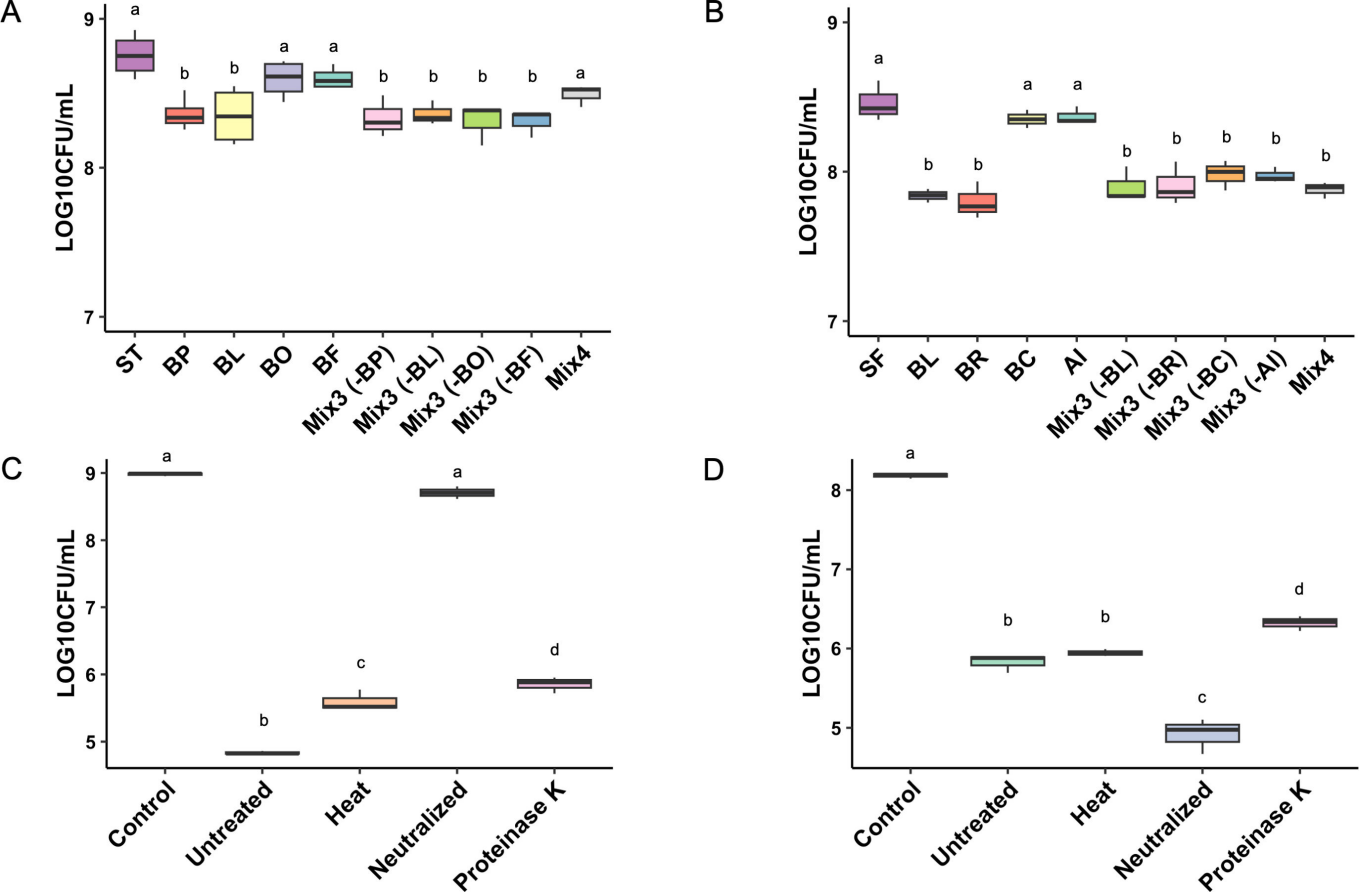
*In vitro* inhibition of pathogens by a synthetic bacterial consortium. Inhibitory effects of bacterial mixes (Mix3 and Mix4) on (**A**) *Salmonella* (ST) and (**B**) *Shigella* (SF). Mixes were composed of selected strains (BP, BL, BO, BF, BR, BC, and AI). BP, *B. producta*; BL, *B. longum*; BO, *B. ovatus*; BF, *B. fragilis;* BR, *B. ruminantium*; BC, *B. circulans*; AI, *A. indistinctus*. Inhibition of (**C**) *Salmonella* and (**D**) *Shigella* by spent media from BL cultured for 48 h under various treatment conditions. Control: pathogen cultures in fresh modified brain heart infusion (mBHI) medium. The experiment was performed in triplicate, and statistical analysis was conducted using ANOVA and Tukey HSD test. Different letters indicate statistically significant differences (*P* < 0.05).

Having identified species with relatively low cell invasiveness that could inhibit both pathogens, we next tested whether additive inhibition could be obtained by a pool of all these species. Based on the pathogen inhibition rank, we selected the best four species and constructed a synthetic consortium. Single-species dropout experiments were also conducted by omitting one to demonstrate the importance of individual species in the consortium. When compared to individual species inhibition capacity, no mix improved the inhibition of *Salmonella* or *Shigella* (Fig. 2A and B), clearly showing that the inhibitory capacity is not additive. Notably, *B. longum* alone significantly reduced the growth of both *Salmonella* and *Shigella* in the co-culture assay, with inhibitory effects indistinguishable from those of bacterial mixes.

Pathogen suppression by commensal bacteria may be attributed to multiple mechanisms, including direct nutritional competition, production of bacteriocins and secondary metabolites, and pH alteration (33–35). To elucidate which of these mechanisms is responsible for suppression, we compared the inhibitory effects of spent media on both *Salmonella* and *Shigella* under various treatment conditions. Spent media of *B. longum* significantly inhibited *Salmonella* growth, resulting in a four-log reduction. The treatment of the spent media with heat or Proteinase K slightly decreased this inhibition, suggesting the involvement of proteins and heat-labile components produced by *B. longum* (Fig. 2C). Organic acid production by probiotics, primarily lactic and acetic acid, creates an acidic environment that is well known to inhibit pathogen growth. To determine whether acidification is responsible for the observed antimicrobial activity, pH adjustment is commonly performed. As expected, neutralizing the pH substantially reduced the inhibitory effect, emphasizing the importance of acidic conditions produced by *B. longum* in inhibiting *Salmonella* growth. In contrast, *Shigella* growth was inhibited by approximately two logs in untreated spent media, with heat treatment resulting in no significant effect on inhibition (Fig. 2D). A slight reduction in the level of inhibition was observed after Proteinase K treatment. Notably, the neutralized spent media exhibited the highest degree of inhibition of *Shigella*, suggesting that a pH-independent component is responsible for the suppression of *Shigella* by *B. longum* spent media. In summary, the spent media results indicate that the mechanism of *Salmonella* inhibition by *B. longum* is pH reduction, whereas *Shigella* inhibition occurs through a protein-mediated mechanism or a heat-stable secondary metabolite.

### *B. longum* colonization reduces *Salmonella* load and enhances pathogen resistance in a *C. elegans* model

**Fig 3:**
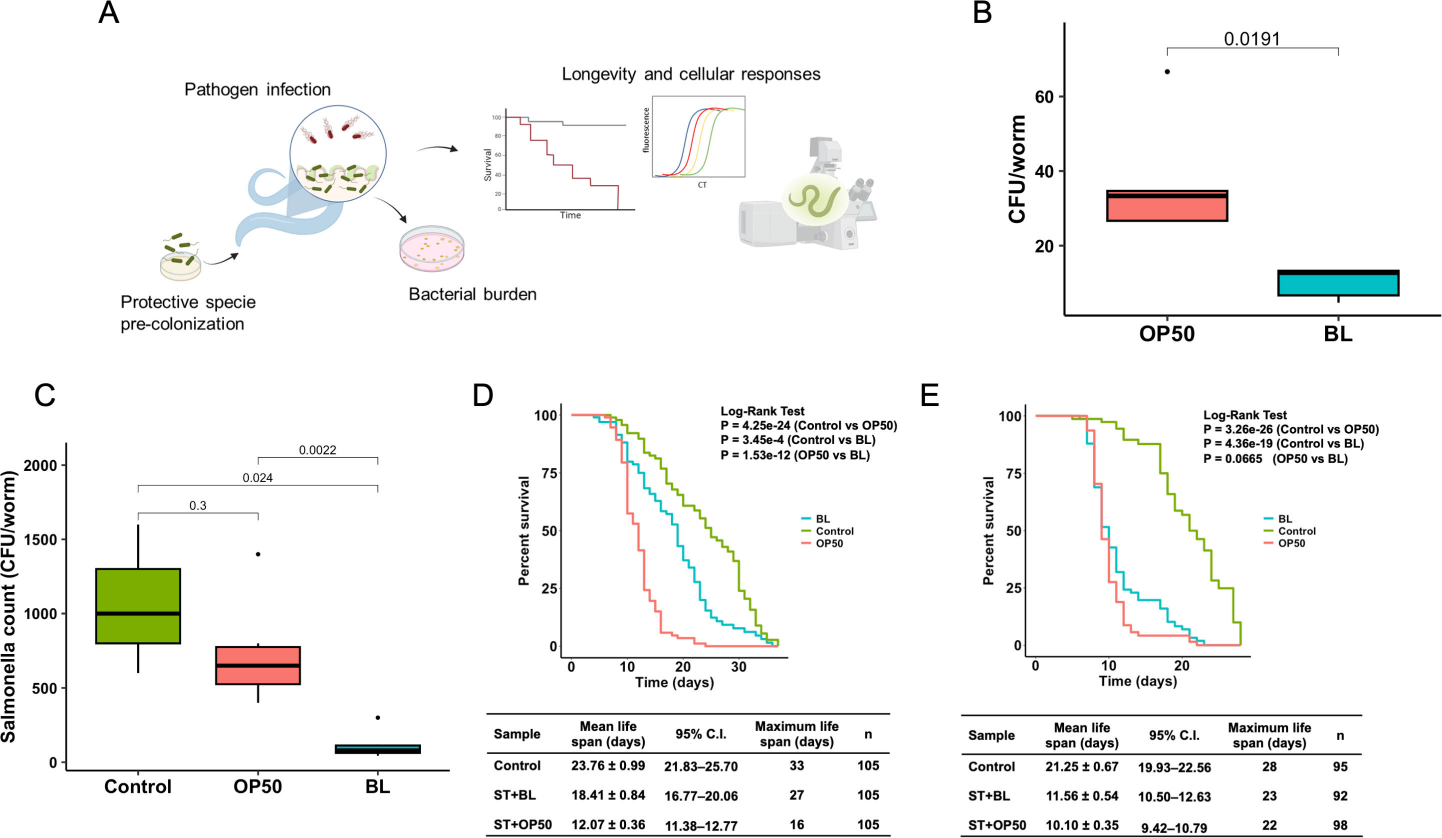
*B. longum* confers protection against *Salmonella* infection in a *C. elegans* model. (**A**) Schematic of the experimental design. Image was created with Biorender. (**B**) Colonization efficiency of *B. longum* (BL) compared to *E. coli* OP50 (OP50) in *C. elegans* 48 h post-feeding. (**C**) *Salmonella* burden in worms precolonized with BL or OP50, measured 48 h post-infection. *C. elegans* pre-colonized with dead OP50 was used as the Control. Survival of precolonized worms following (**D**) acute or (**E**) chronic *Salmonella* (ST) exposure. Lifespan assessed by Kaplan–Meier analysis (log-rank test). The mean lifespan represents the mean value ± standard deviation of worms from different treatments. Control; no ST infection worms.

To determine whether pathogen suppression by *B. longum* observed *in vitro* could be replicated *in vivo*, an animal experiment was conducted using *C. elegans*. Worms were pre-colonized with *B. longum* and subsequently infected with *Salmonella* to assess bacterial load and the effects on worm lifespan (Fig. 3A). *B. longum* successfully colonized and persisted in *C. elegans* following a 2-day pre-colonization period (Fig. 3B). However, the quantity of *B. longum* was significantly lower than that of *E. coli* OP50 (OP50), which is conventionally used as feed for *C. elegans*. As demonstrated in Fig. 3C, a 3 h exposure period was sufficient for *Salmonella* to successfully colonize and induce acute infection in *C. elegans*. Pre-colonization of *C. elegans* with *B. longum* resulted in significantly enhanced *Salmonella* clearance, achieving a one-log reduction compared with worms fed OP50 and the control (dead OP50). The survival of worms infected with *Salmonella* was evaluated under two distinct conditions. Continuous feeding of the worms with *Salmonella* was conducted to replicate chronic exposure, a standard procedure for assessing the pathogen’s ability to kill the worms, and acute exposure by introducing *C. elegans* to the pathogen for 3 h before transferring them to a pathogen-free environment to simulate the normal course of infection. Both continuous and brief exposure to *Salmonella* significantly affected the lifespan of worms (Fig. 3D and E). Colonization with *B. longum* results in substantial protection against *Salmonella* during acute infection. The percent survival of *C. elegans* pre-colonized with *B. longum* was significantly higher (*P* < 0.001) with a mean life span of 18.41 ± 0.84 days, compared to 12.07 ± 0.36 days in the worms fed with OP50 (Fig. 3D). However, no protective effect of *B. longum* was observed when *C. elegans* was continuously exposed to *Salmonella*. In conclusion, *B. longum* had a protective effect against acute *Salmonella* infection in a *C. elegans* model, and this protective mechanism appears to be independent of niche competition. Furthermore, the inhibitory effect of *B. longum* could potentially be extended beyond non-typhoidal strains to include typhoidal strains, thereby broadening its therapeutic applications.

### *Shigella* reduces worm life span, and *B. longum* increases longevity

**Fig 4:**
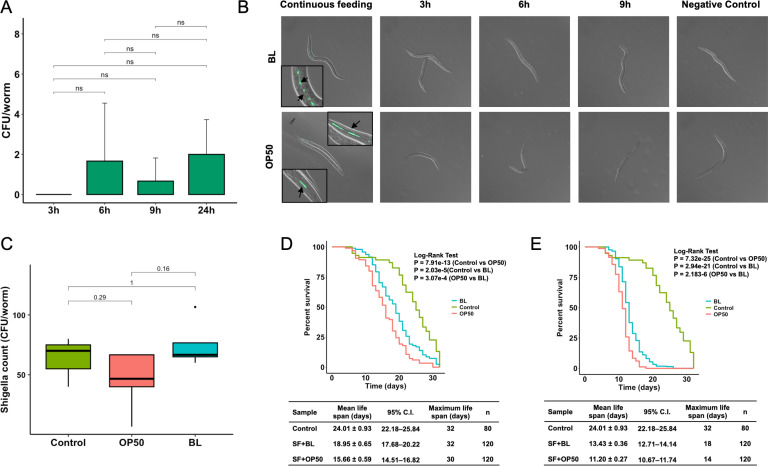
*B. longum* enhances *C. elegans* survival during *Shigella* infection. (**A**) *Shigella* load in worms exposed for 3 h, 6 h, 9 h, or 24 h, quantified 48 h post-infection (ANOVA, Tukey HSD; *P* < 0.05). (**B**) Confocal imaging of GFP-labeled *Shigella* in worms precolonized with *B. longum* (BL) or *E. coli* OP50 (OP50). (**C**) *Shigella* burden in worms pre-colonized with BL or OP50, assessed 48 h after continuous exposure. *C. elegans* pre-colonized with dead OP50 served as the Control. Survival of precolonized worms following (**D**) acute or (**E**) chronic *Shigella* (SF) exposure. Lifespan assessed by Kaplan–Meier analysis (log-rank test). The mean lifespan represents the mean value ± standard deviation of worms from different treatments. Control; no SF infection worms.

To determine the effect of *Shigella* on *C. elegans* and the potential protective role of *B. longum*, analogous to the experimental model employed for *Salmonella*, both acute and chronic exposure of *C. elegans* to *Shigella* were investigated. Initially, we were unable to quantify *Shigella* loads in *C. elegans* transiently exposed to the pathogen, in contrast to those that were continuously exposed. This observation led us to postulate that *Shigella* does not persist in the intestinal tract of *C. elegans*. To test this hypothesis, we examined *Shigella* colonization in *C. elegans* by varying the exposure duration from 3 h to 24 h before transferring the worms to a pathogen-free environment. Colony-forming units (CFU) were assessed once the worms reached the young-adult stage. As anticipated, we were unable to observe any CFU of *Shigella* from *C. elegans*, irrespective of the exposure period (Fig. 4A). The inability of the pathogen to persist in *C. elegans* was further corroborated by visualization of GFP-tagged bacteria. No GFP signal was detected after temporary feeding of *C. elegans* with bacteria for 3 h, 6 h, or 9 h, supporting our findings from the previous set of experiments (Fig. 4B). Consequently, *Shigella* burden in *C. elegans* was assessed under chronic exposure conditions. In contrast to *B. longum’s* protection against *Salmonella*, the *Shigella* load in the worms was comparable across all treatments (Fig. 4C), suggesting that colonization by *B. longum* is insufficient to reduce the *Shigella* burden in *C. elegans*. Despite the inability of *Shigella* to persist in *C. elegans*, worm longevity was significantly reduced following both acute and chronic exposure to the pathogen. This indicates that stable colonization by *Shigella* is not necessary to induce worm mortality. The observed effect may be driven by colonization-independent mechanisms, such as toxin-mediated damage (e.g., Shiga toxin or enterotoxins) or stress-induced responses to pathogen exposure. Further studies are needed to validate these possibilities and elucidate the underlying mechanisms of host mortality. The survival assay of *C. elegans* under acute bacterial infection demonstrated a statistically significant difference in worm life span (*P* < 0.001), with the mean life span extending from 15.66 ± 0.56 days in OP50-fed worms to 18.95 ± 0.65 days in worms pre-colonized with *B. longum* (Fig. 4D). The capacity of *B. longum* to enhance pathogen suppression in *C. elegans* was also demonstrated under chronic exposure conditions, as evidenced by the increased mean lifespan of 13.43 ± 0.36 days in *C. elegans* pre-colonized with *B. longum* compared to those fed with OP50 (11.20 ± 0.27 days). These results suggest that exposure to *Shigella* significantly affects worm longevity independently of pathogen colonization, whereas *B. longum* colonization enhances worm longevity.

### *B. longum* causes suppression of genes and pathways associated with the host defense during *Salmonella* and *Shigella* infections

**Fig 5:**
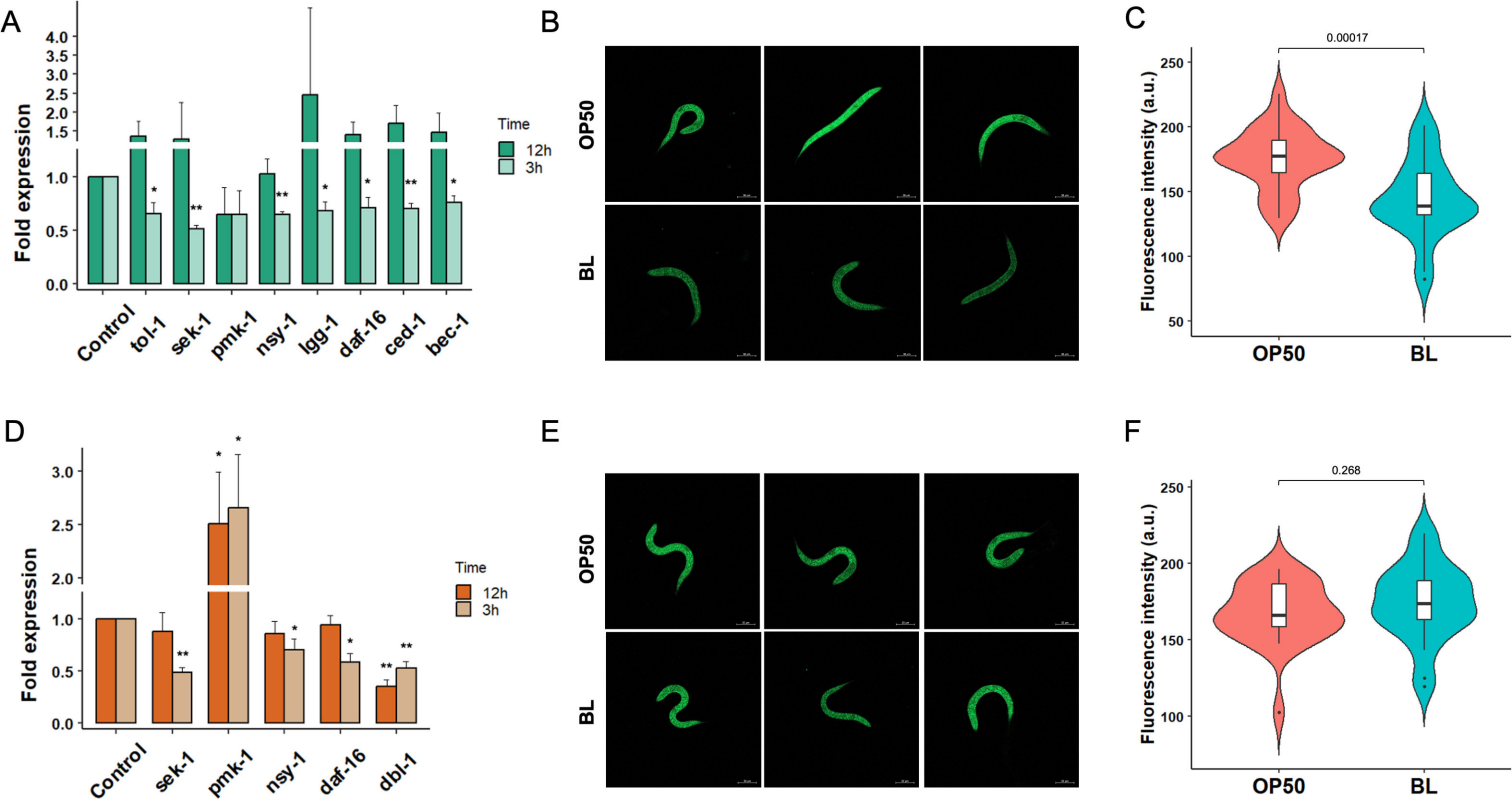
Suppression of innate immunity indicates the protective effects of *B. longum* against pathogenic infections in *C. elegans*. Differential gene expression in *C. elegans* upon exposure to (**A**) *Salmonella* and (**D**) *Shigella* at 3 and 12 h post-infection. Gene expression was quantified by qRT-PCR and normalized to that of the housekeeping gene *pmp-3*. Expression of DAF16::GFP in CF1139 transgenic *C. elegans* mutants in response to (**B and C**) *Salmonella* or (**E and F**) *Shigella* at 3 h post-infection, compared between worms fed *B. longum* (BL) to those fed *E. coli* OP50 (OP50). Worms (*N* = 25) from three biological replicates were used for the quantitative analysis of fluorescence intensity using FIJI software. Statistical differences between groups were analyzed using Student *t*-test (*P < 0.05 and **P < 0.01).

Previously, we reported that the colonization of *C. elegans* with *B. longum* resulted in the upregulation of innate immune and age-associated genes (36). To investigate differential host responses to *Salmonella* and *Shigella* infections, the genes and pathways associated with host defense were analyzed in *C. elegans* using qRT-PCR. Three hours post-infection, downregulation of the p38 MAPK pathway was observed in *C. elegans* pre-colonized with *B. longum*, as evidenced by the reduced levels of SAPK/ERK kinase-1 (*sek-1*) and neuronal symmetry (*nsy-1*) compared with those in worms fed OP50. Relative to OP50 feeding, colonization with *B. longum* resulted in the suppression of forkhead box protein O (*daf-16*), LC3, GABARAP and GATE-16 family (*lgg-1*), and beclin homolog 1 (*bec-1*), leading to the attenuation of autophagy in *C. elegans* in response to *Salmonella* infection. Additionally, a decrease in the expression of toll-like receptor (*tol-1*) and cell death abnormality protein 1 (*ced-1*), which are related to apoptosis in *C. elegans*, was observed (Fig. 5A). No significant changes in gene expression were observed 12 h post-infection. The *C. elegans* transgenic mutant CF1139 expressing DAF16::GFP was used to confirm the expression level of DAF16 using confocal microscopy. A relatively lower GFP signal was detected in CF1139 worms pre-colonized with *B. longum* during *Salmonella* infection than in OP50 feeding (Fig. 5B and C). Therefore, it appears that the protective effect of *B. longum* against *Salmonella* infection in *C. elegans* occurs through host immune modulation, which could be indicated by the downregulation of host defense genes related to pathogen infection. We also analyzed the differences in gene expression related to the host response in *C. elegans* pre-colonized with *B. longum* compared with OP50 during *Shigella* infection (Fig. 5D). Significant reductions in the expression of genes and pathways contributing to the immune response, including *sek-1*, *nsy-1*, *daf-16*, and Dpp and BMP-like protein 1 (*dbl-1*), were observed 3 h post-infection. While most responses were restored 12 h post-infection, the repression of dbl-1 remained evident. Our findings demonstrate that *B. longum* attenuates *Shigella* infection by suppressing genes involved in the innate immune response of *C. elegans*. Despite changes in mRNA levels, no difference in DAF16::GFP expression was observed in CF1139 worms (Fig. 5E and F ).

## DISCUSSION

Using a previously established healthy human microbiota culture collection (20), we conducted a high-throughput screen to identify strains capable of inhibiting *S*. Typhimurium and *S. flexneri. In vitro* co-culture assays revealed pathogen-inhibiting, inhibition-neutral, and pathogen growth-enhancing strains. Most strains tested against *Salmonella* showed mild inhibition or had no effect on pathogen growth. Notably, the inhibiting strains primarily belonged to the genus *Bifidobacterium*, while numerous moderately inhibitory strains were from the genus *Bacteroides*. Similar trends were observed with *Shigella*. However, a key difference was that many strains markedly increased the abundance of *Shigella*, several of which were members of *Bacteroidetes*. This unanticipated finding aligns with the known trends in gut ecology. Pathogens typically exist in low numbers in the mammalian because the diverse microbial community prevents their overgrowth. However, gut dysbiosis can disrupt this balance, facilitating both pathogens and many co-colonizing species to proliferate (37). *Bacteroides thetaiotaomicron*, a known gut symbiont that aids dietary sugar degradation and improves gut maturation (38), can also worsen infections by enterohemorrhagic *E. coli* (39). It should be noted that the outcome of inhibition assays may be influenced by the ratio of the bacterial strain to the pathogen. Further investigation into dose-dependent effects would provide additional justification for these interactions.

*B. longum* and *E. coli* showed strong inhibition against both *Salmonella* and *Shigella*. Due to the invasiveness of the *E. coli* strain, only *B. longum* was utilized for subsequent mechanistic investigations. Synthetic mixtures comprising other inhibiting strains, with and without *B. longum*, did not enhance the inhibition when compared to that of *B. longum* alone. This outcome was unexpected, as additive inhibitory effects have been observed in multiple species combinations against enteric pathogens like *Clostridium difficile* (20, 40, 41). The lack of additive or enhanced inhibition observed in synthetic mixtures, compared to individual strains, may suggest potential competition among community members. This could result from antagonistic interactions, such as interference from strain-specific metabolites, or niche saturation that limits the overall carrying capacity and population growth. These factors may reduce the collective inhibitory effect, highlighting the complexity of designing effective multi-strain consortia. The ability of a single gut commensal to inhibit multiple enteric pathogens has been reported only to a limited extent. Our results demonstrate that *B. longum* possesses dual-pathogen inhibition capacity, highlighting its potential as a broad-spectrum probiotic candidate. To better understand the factors responsible for inhibition, we analyzed the effects of the spent supernatant. *Salmonella* inhibition appeared to be driven by acidification, as buffered supernatants lost most of their inhibitory capacity. In contrast, *Shigella* inhibition was reduced by heat and proteinase K treatment, indicating involvement of heat-stable metabolites or proteinaceous components. Bacteria within the *Bifidobacterium* genus are known to produce a variety of antimicrobial metabolites, including short-chain fatty acids and bacteriocins. Therefore, the *B. longum* strain likely employs a combination of these mechanisms to inhibit pathogenic bacteria (42, 43). Further characterization of these components using proteomic and metabolomic analyses is necessary.

Although the *in vitro* screen provided key initial insights, it did not account for host-related factors such as immune responses and other host defense mechanisms. To ensure the safety of the probiotic candidate, we confirmed that the *B. longum* strain, obtained from a healthy human fecal sample, was non-invasive in Caco-2 cell assays. In addition, *B. longum* has been extensively studied and is generally recognized as safe in mammalian models (44, 45). Following this, *C. elegans* was used in an *in vivo* model to further assess host-microbe interactions and probiotic effects. This worm is widely used in microbiome research due to its short life cycle, ease of handling, and well-characterized immune responses (46, 47). *Salmonella* infections, both acute and chronic, significantly reduced worm lifespan. However, *B. longum* extended survival in the acute infection model, suggesting a protective effect. No protection was observed in the chronic model. This may be due to a differing ratio of *B. longum* to the pathogen (48). During acute infection, limited exposure (3 h) may have allowed pre-colonized *B. longum* to effectively inhibit *Salmonella* proliferation. In continuous exposure, increased pathogen burden may have overwhelmed *B. longum*’s protective effect. Pathogen count and fluorescence images revealed that *Shigella* was unable to colonize and establish a persistent infection in *C. elegans*. The worm grinder likely disrupts ingested *Shigella* (49, 50). The virulence plasmid has been reported to be essential for *S. flexneri* accumulation in *C. elegans* (51), and the strain used in our study may have lacked this component, which could explain its inability to colonize the worm intestine. *S. flexneri* was selected in this study due to its well-characterized pathogenesis, making it a suitable representative for investigating bacterial infection and potential intervention strategies. Although *B. longum* did not reduce *S. flexneri* burden in *C. elegans* model, it appeared to enhance host survival following infection. Future studies evaluating the protective effect of *B. longum* against other clinically relevant *Shigella* species would provide more insight into the protective capabilities of this strain.

Analysis of *C. elegans* host defense and innate immune genes revealed significant modulation of these genes during *B. longum* colonization and pathogen infection. *Salmonella* infection is known to trigger p38 MAPK signaling pathway in *C. elegans* (52). Our RT-qPCR results confirmed that *B. longum* colonization suppressed expression of p38 MAPK-associated genes after 3 h of *Salmonella* infection, compared to worms fed with OP50. Reduced mRNA levels of *lgg-1*, *bec-1*, and *daf-16* suggest altered regulation of genes involved in the autophagic response, a key mechanism for *C. elegans* to defend against various pathogens, including *Salmonella* (53). This is further supported by the results of fluorescent-labeled proteins, as the process is activated under the regulation of DAF-16. Furthermore, the downregulation of *tol-1*, a gene predicted to be involved in pathogen recognition (54), was also observed, supporting the finding that *B. longum* enhances pathogen clearance.

Compared to *Salmonella*, *Shigella* has been relatively less studied in *C. elegans*. Therefore, we analyzed genes involved in p38 MAPK, DBL-1/TGF-β, and the insulin-like signaling pathway, which are key regulators of intestinal defense (55). As hypothesized, the expression of immune-related genes was predominantly downregulated in *B. longum*-colonized worms compared to those fed OP50 following *Shigella* infection. Conversely, we observed an increase in *pmk-1* expression levels at 3 h and 12 h post-infection, potential crosstalk between p38 MAPK and other pathways through PMK-1 regulation (56, 57). Our recent study reported that feeding *C. elegans* with *B. longum* extends worm longevity and alters the expression of genes associated with autophagy and lysosomal functions (36). This supports the idea that autophagy may contribute to *B. longum*-mediated colonization resistance. It should be noted that changes in mRNA levels do not always correlate with protein function; therefore, future experiments focusing on protein-level analyses, such as proteomic studies or reporter-based assays, are necessary to validate these findings. While *C. elegans* is a valuable model for studying probiotics and host-microbe interactions, its lack of an adaptive immune system and simplified circulatory system may limit its ability to fully demonstrate the complex immune responses seen in higher organisms. This absence restricts the model’s translational relevance, particularly for understanding interactions that involve adaptive immunity mechanisms. Therefore, further validation in mammalian models, such as studies of colonization resistance in gnotobiotic systems, is essential to provide a more comprehensive assessment of probiotic effects and host responses.

**Fig 6:**
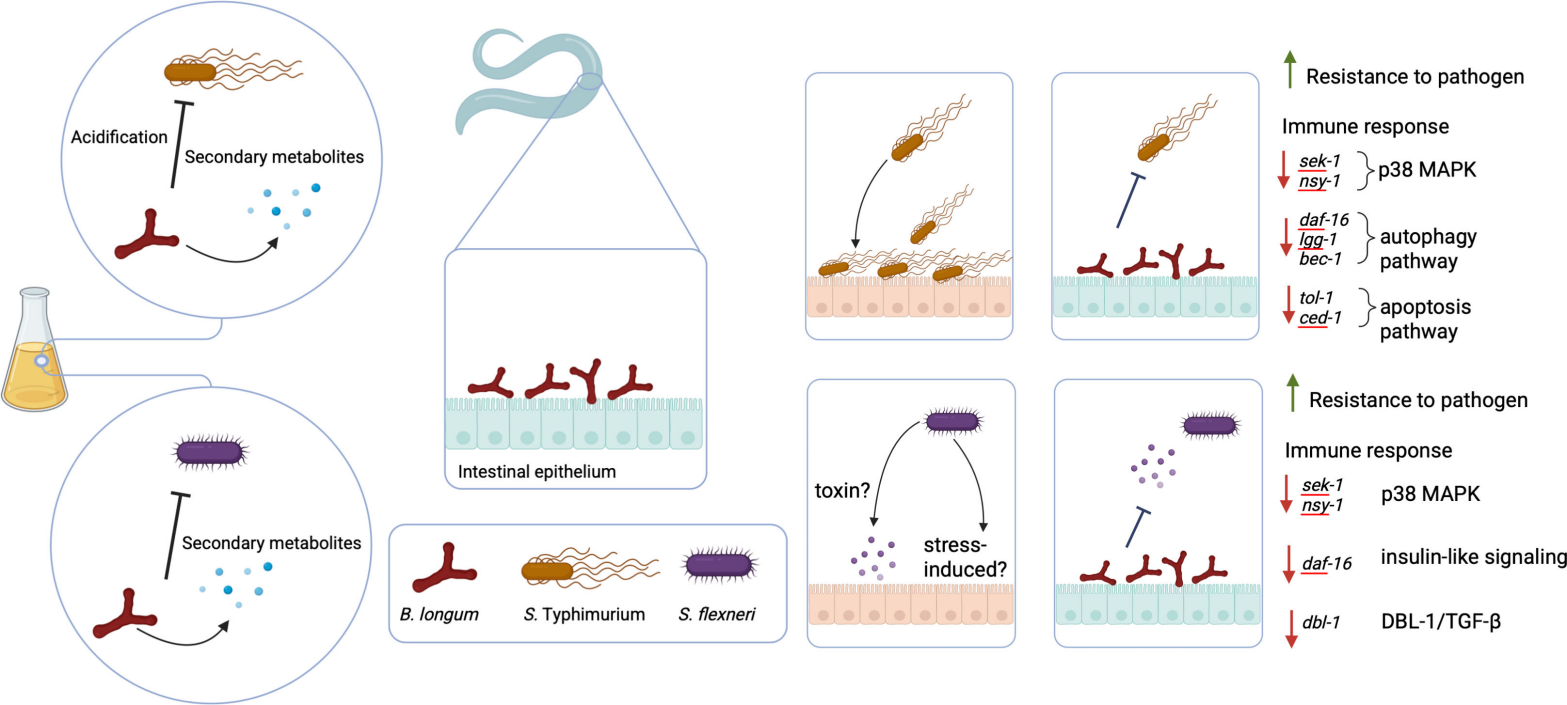
Summary of the pathogen-inhibitory effect of *B. longum* and its contribution to colonization resistance in *C. elegans*, potentially through modulation of immune pathways. The image was created with Biorender.

Our findings reveal that *B. longum*, isolated from the human gut, can inhibit the growth of *Salmonella* and *Shigella* (Fig. 6). The acidic environment created by *B. longum* significantly impairs *Salmonella* growth, whereas suppression of *Shigella* likely involved a secreted protein or heat-stable metabolite. Additionally, *B. longum* colonization modulated innate immune pathways during pathogen exposure, indicating its potential to attenuate the severity of infection. While *B. longum* has potential as a non-antibiotic probiotic candidate, additional studies in mammalian models are required to validate these findings and evaluate their clinical relevance.

## MATERIALS AND METHODS

### Bacterial strains and culture conditions

The bacterial species used in this study were selected from a human gut culture library previously constructed in our laboratory (20). All species were cultured and maintained in modified brain heart infusion (mBHI) medium at 37°C under anaerobic conditions with 85% CO_2_, 10% H_2_, and 5% N_2_. *Salmonella* Typhimurium 4,[5],12:i:, isolated from swine samples (58), and *S. flexneri* ATCC 12022 were cultured in Luria-Bertani medium. Xylose Lysine Tergitol 4 (XLT4) agar (BD Difco, Houston, TX, USA) was used for the selective identification of *Salmonella* and *Shigella*.

### High-throughput screening and co-culture assays

Screening of bacterial strains capable of inhibiting *S*. Typhimurium and *S. flexneri* was performed using a coculture assay. Overnight cultures of bacteria in mBHI broth were adjusted to an OD_600_ of 0.5, and co-cultivation with the pathogen was performed at a ratio of 9:1 in a final volume of 1 mL mBHI. After 24 h of incubation at 37°C in an anaerobic environment, the bacterial suspensions were serially diluted 10-fold in phosphate-buffered saline (PBS), and pathogen counts were enumerated using the drop plate method on XLT4 agar. Cultures containing only *S*. Typhimurium or *S. flexneri* were used as untreated controls. The degree of pathogen inhibition was assessed by comparing colony counts to the control after 24 h. For combination mixtures, individual strains adjusted to 0.5 OD_600_ were mixed to create Mix3 or Mix4 and tested in the co-culture assay with a consistent ratio of 9:1.

### Caco-2 cell invasion assay

Human colorectal adenocarcinoma (Caco-2) cells were used to evaluate the invasive potential of the bacterial isolates. Overnight cultures of each bacterial strain were used to infect Caco-2 cells at a multiplicity of infection of 1:100 and incubated for 1 h at 37°C. Following infection, the bacterial suspensions were removed, and the cells were washed twice with PBS. A Dulbecco's modified eagle medium (DMEM) solution supplemented with 100  µg/mL gentamicin was added to the cells and incubated for an additional hour to ensure the elimination of extracellular bacteria. Subsequently, the cells were lysed with 1% Triton X-100 under anaerobic conditions, and intracellular bacteria were quantified by CFU counts on mBHI agar.

### Spent media assay

To prepare the spent medium, *B. longum* was cultured in mBHI broth for 48 h to reach the stationary phase. The culture was then collected by centrifugation and filtered through a 0.22 µm filter to remove residual bacterial cells. The resulting filtrate was divided into four portions, each subjected to different treatment conditions. Heat treatment involved heating the filtrate to 90°C for 1 h to preserve heat-stable components while inactivating heat-labile compounds. For protein removal, the filtrate was incubated with Proteinase K (1 mg/mL) for 2 h. To obtain neutralized spent media, the pH of the filtrate was adjusted to ~7.0 with NaOH, followed by a second filtration through a 0.22 µm filter. The inhibition efficacy of the treated spent media was evaluated by incubating 0.5 OD_600_ of *Salmonella* or *Shigella* with 500 µL of the treated media under anaerobic conditions at 37°C for 24 h. Pathogen growth was assessed by CFU on XLT4 agar plates. A negative control was performed using fresh mBHI broth.

### Bacterial colonization and pathogen infection in *C. elegans*

Wild-type N2-Bristol *C. elegans* were maintained on nematode growth medium (NGM) plates (RPI, Mt Prospect, IL) seeded with *E. coli* OP50 at 20°C. Synchronization was achieved via bleach treatment, allowing eggs to hatch for 14–16 h to obtain synchronized L1 stage worms. Bacterial colonization was performed as described previously (36). Briefly, synchronized L1 worms were exposed to a concentrated lawn of *E. coli* OP50 or *B. longum* in an anaerobic chamber for 3 h. Following exposure, worms were transferred to a biosafety cabinet for aeration for an additional hour. Worms were then washed three times with M9 buffer and transferred to an NGM plate seeded with dead OP50 prepared as previously described (59). After a 2-day colonization period, 20–30 worms were picked and surface-sterilized using a 1:1,000 bleach solution in M9 buffer. The worms were washed three times and resuspended in anaerobic PBS before being lysed with a motorized pestle in the anaerobic chamber. The number of *B. longum* colonies in *C. elegans* was determined by CFU counts on mBHI agar. To investigate the protective effect against pathogens, *C. elegans* pre-colonized with *B. longum* or OP50 for 24 h were briefly exposed to a concentrated pathogen for 3 h. Following exposure, worms were surface-sterilized and washed three times before being transferred to NGM plates seeded with a lawn of dead OP50. Pathogen load was determined by counting CFU on XLT-4 agar plates.

### *C. elegans* survival assay

For short-term exposure to pathogens, 30–40 pre-colonized L3/L4 worms were fed a concentrated lawn of *Salmonella* or *Shigella* for 3 h. The worms were then surface sterilized and washed three times before being transferred to fresh NGM plates seeded with dead OP50. To evaluate the protective effect under continuous pathogen exposure, worms were constantly fed pathogens on NGM plates. The number of dead worms was recorded daily, and worms that crawled off the plates were excluded from the analysis.

### RNA isolation and qRT-PCR

Total RNA was extracted using the TRIzol reagent (Thermo Fisher Scientific, Waltham, MA, USA). Worms were snap frozen in liquid nitrogen and subjected to multiple freeze-thaw cycles to ensure complete lysis. Reverse transcription was performed using ProtoScript II Reverse Transcriptase (New England BioLabs), following the manufacturer’s instructions. The resulting cDNA was used for qRT-PCR using the Power SYBR Green PCR Master Mix (Applied Biosystems, Waltham, MA, USA). The peroxisomal membrane protein-related gene (*pmp-3*) was used as the reference gene, and fold changes in gene expression were calculated using the 2^−ΔΔCt^ method.

### *C. elegans* fluorescence imaging and quantification

To evaluate *Shigella* burden in *C. elegans*, L3/L4 worms were temporarily fed *Shigella-*GFP for 3 h, 6 h, or 9 h, then transferred to NGM plates seeded with a lawn of dead OP50. Worms at the young adult stage were surface sterilized, washed three times, and visualized under a confocal microscope (Zeiss LSM 980). The DAF16::GFP-expressing worm strain CF1139 was used to assess gene expression. Worms expressing L3/L4 GFP were briefly exposed to pathogens for 3 h. Three hours post-infection, the worms were fixed with 4% paraformaldehyde in PBS, washed three times, and visualized using a confocal microscope. Quantification of the GFP signal was performed using the FIJI analysis tool (ImageJ).

## SUPPLEMENTAL MATERIAL

Figure S1 spectrum.01842-25-s0001.docx Invasion of Caco-2 cells by various bacterial strains. The ability of different bacterial species to invade Caco-2 epithelial cells was assessed using a gentamicin protection assay. Invasive bacteria were quantified as LOG10CFU/ml recovered from within Caco-2 cells. Values represent the mean from three independent experiments.

ASM does not own the copyrights to Supplemental Material that may be linked to, or accessed through, an article. The authors have granted ASM a non-exclusive, world-wide license to publish the Supplemental Material files. Please contact the corresponding author directly for reuse.
