# Supplementary material for: Gut commensal Bifidobacterium longum confers resistance to Salmonella Typhimurium and Shigella flexneri in a Caenorhabditis elegans model
Source: Microbiol Spectr. 2025 Dec 5;14(1):e01842-25. doi: 10.1128/spectrum.01842-25 (PMC12772369; doi:10.1128/spectrum.01842-25)
Supplement: Figure S1 — Invasion of Caco-2 cells by various bacterial strains. The ability of different bacterial species to invade Caco-2 epithelial cells was assessed using a gentamicin protection assay. Invasive bacteria were quantified as LOG10CFU/ml recovered from within Caco-2 cells. Values represent the mean from three independent experiments. [file spectrum.01842-25-s0001.docx]

**SUPPLEMENTARY MATERIAL**

**
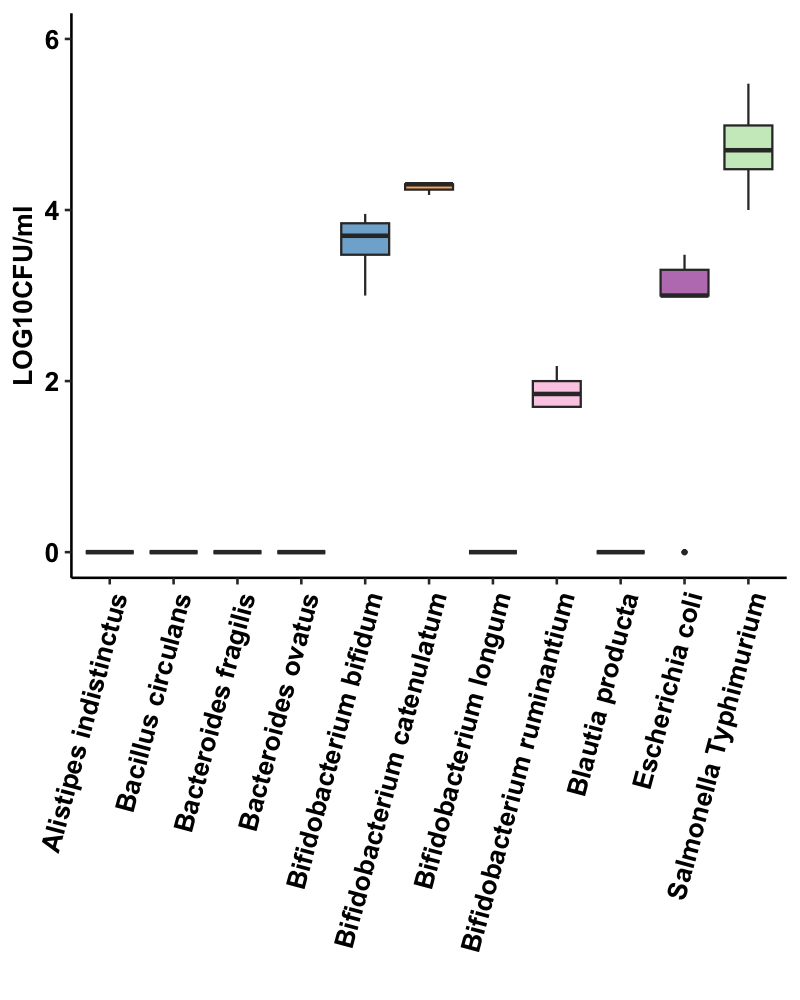
**

**Supplementary Figure 1.** Invasion of Caco-2 cells by various bacterial strains. The ability of different bacterial species to invade Caco-2 epithelial cells was assessed using a gentamicin protection assay. Invasive bacteria were quantified as LOG10CFU/ml recovered from within Caco-2 cells. Values represent the mean from three independent experiments.
